# circGNB1 Facilitates Triple-Negative Breast Cancer Progression by Regulating miR-141-5p-IGF1R Axis

**DOI:** 10.3389/fgene.2020.00193

**Published:** 2020-03-05

**Authors:** Peng Liu, Yutian Zou, Xing Li, Anli Yang, Feng Ye, Jie Zhang, Weidong Wei, Yanan Kong

**Affiliations:** Department of Breast Oncology, Sun Yat-sen University Cancer Center, State Key Laboratory of Oncology in South China, Collaborative Innovation Center for Cancer Medicine, Guangzhou, China

**Keywords:** circGNB1, circular RNAs, IGF1R, competitive endogenous RNAs, triple negative breast cancer

## Abstract

As an intriguing class of RNA, circular RNAs (circRNAs) are vital mediators of various diseases including cancers. However, the biological role and underlying mechanism of the majority of circRNAs are still ambiguous in the progression of triple-negative breast cancer (TNBC). In this study, we characterized and further investigated hsa_circ_0009362 (circGNB1) by reanalyzing the circRNA microarray profiling in our previous study. Validating by qRT-PCR, circGNB1 was overexpressed in TNBC cell lines and high expression of circGNB1 was associated with worse clinical features and survival outcomes. The expression of circGNB1 was positively correlated with tumor size and clinical stage, and high expression of circGNB1 was an independent risk factor for TNBC patients. Cell proliferation, colony formation, wound-healing and mouse xenograft assays were carried out to investigate the functions of circGNB1. Both *in vitro* and *in vivo* assays revealed that knockdown of circGNB1 significantly suppressed cell proliferation, migration and tumor growth. Subsequently, we performed luciferase reporter assays and RNA immunoprecipitation assays to elucidate the underlying molecular mechanism of circGNB1. The results showed that circGNB1 sponges miR-141-5p and facilitates TNBC progression by upregulating IGF1R. Altogether, our study demonstrated the pivotal role of circGNB1-miR-141-5p-IGF1R axis in TNBC growth and metastasis though the mechanism of competing endogenous RNAs. Therefore, circGNB1 may have the potential to be a therapeutic target and novel prognostic biomarker for TNBC.

## Introduction

According to the global estimated cancer statistic, breast cancer is the most common malignancy and second major cause of cancer-related deaths among women worldwide (Bray et al., 2018). Regarded as a high heterogeneous disease, breast cancer can be divided into four major different molecular subtypes (Harbeck and Gnant, 2017). Among these four subtypes, triple-negative breast cancer (TNBC) is characterized by the loss of expression of human epidermal growth factor receptor 2 (HER2) and hormonal receptors, which accounts for approximately 15% of all breast cancers (Carey et al., 2010). Lacking of effective therapeutic target, TNBC has the highest metastatic rate and worst prognosis which occurs more frequently in young women (Foulkes et al., 2010; Xiao et al., 2018a). Therefore, it is urgent to elucidate the underlying mechanisms which contribute to the progression of TNBC and identify new treatment strategies for patients with TNBC.

In recent years, circular RNAs (circRNAs) were widely studied in the fields of the life sciences for its various biological functions in cells. By regulating the expression of key genes, circRNAs play important roles in the development and progression process of different kind of cancer (Kristensen et al., 2018). As a type of endogenous non-coding RNAs (ncRNAs), circRNAs are widely present and expressed in mammalian cells with a linear structure (Jeck et al., 2013). circRNAs are highly conversed and formed by the back splicing of exons or introns without a 5′-cup or 3′-poly A tail, which are more stable and abundant than linear mRNAs (Zhang et al., 2014). Once were regarded as the byproducts of splicing errors, circRNAs are mediators of cell biological activities by versatile mechanisms, including sponging microRNA (miRNA), binding proteins and encoding novel small proteins (Li et al., 2018). RNAs (mRNAs, long non-coding RNAs, circRNAs and etc.) can serve as competing endogenous RNAs (ceRNAs) and communicate with each other by binding miRNAs, according to the ceRNA hypothesis (Salmena et al., 2011; Tay et al., 2014). Accumulating evidence indicates that circRNAs regulate the cell biological process by acting as miRNA sponges. For example, the most well-known circRNA CDR1as promotes proliferation and invasion of different tumors by blocking miR-7 (Hansen et al., 2013b; Weng et al., 2017; Pan et al., 2018; Zou et al., 2019a). circFAT1 suppresses gastric cancer progression by sponging miR-548g and upregulates RUNX1 tumor suppressor (Fang et al., 2019). In previous study, circKIF4A, circRAD18 and circPLK1 were also identified and proved to be oncogenic regulators in the progression of breast cancer by interacting with miRNAs (Kong et al., 2019; Tang et al., 2019; Zou et al., 2019b). Despite the progress and advancements in the study of circRNAs, the potential functions and the underlying molecular mechanism of the most circRNAs are still remained unclear.

In the present study, we identified a frequently upregulated novel circRNA (hsa_circ_0009362, circGNB1) in TNBC by analyzing our previous circRNA microarray profiling. A series of experiments and bioinformatic analysis were conducted to study the biogenesis, functions and mechanisms of circGNB1 in TNBC. Generally, our study demonstrated the pivotal role of circGNB1-miR-141-5p-IGF1R axis in TNBC growth and metastasis though the mechanism of competing endogenous RNAs. Thus, circGNB1 may have the potential to be a therapeutic target and novel prognostic biomarker for TNBC.

## Materials and Methods

### Clinical Data and Patient Samples

Fresh breast cancer samples were collected from patients at Sun Yat-sen University Cancer Center (SYSUCC, China). All the resected breast cancer tissues were immediately infiltrated into RNAlater reagent (Ambion, Texas). This study was approved by the Ethics Committee of Sun Yat-sen University Cancer Center Health Authority and conducted in accordance with the Declaration of Helsinki. Written informed consent was collected from all patients before participation in this study.

### Cell Culture

All cell lines (MCF-10A, MDA-MB-231, BT549, HCC1806, HCC38, MCF-7, T47D, BT474, SKBR-3, and MDA-MB-361) used in this study were obtained from the ATCC. Being cultured and passaged for less than 6 months, all of the above cell lines were proofed free of mycoplasma infection verifying occasionally by DNA fingerprinting.

### qRT-PCR Analysis

Total RNA of cells and samples were extracted by TRIzol reagent (Invitrogen). The cytoplasmic and nuclear RNA in the cells were isolated using NE-PER Nuclear and Cytoplasmic Extraction Reagents (Thermo Fisher Scientific). qRT-PCR analysis was performed using SYBR Premix Ex Taq (Takara). The sequences of primers used in qRT-PCR analysis is listed in Supplementary Table S2.

### Cell Oligonucleotide Transfection

Transfection of MDA-MB-231 and BT-549 cells was performed with Lipofectamine 3000 (Invitrogen). The miRNA mimics and inhibitors were provided by GeneCopoeia (Rockville). The small interfering RNAs (siRNAs) were synthesized by RiboBio (Guangzhou,China).

### CCK-8 Assay

The transfected cells were seeded into the well of a 96-well plate at a density of 2 × 10^3^ and cultured for 2 days. Ten microliters of CCK-8 reagent (Dojindo Corp, Japan) was added to each well. After incubation for 2 h at 37°C, the absorbance at a wavelength of 450 nM was measured using a microtiter plate reader.

### Colony Formation Assay

A total of 1 × 10^3^ cells were resuspended and replanted into a 6-well plate. After incubation for 10 days in an appropriate condition, the cell colonies were fixed with methanol and stained with 0.3% crystal violet for half an hour. Images were obtained right away after staining. Image J software was utilized to count and recorded the number of colonies in each well.

### Wound Healing Assay

Generally, cells were cultured in 6-well plates. At least three artificial linear wounds were made by scratching with a 200 μL pipette tip and the position was marked afterward. At the time period of 0 and 24 h, each wound was imaged with an inverted microscope.

### Dual Luciferase Reporter Assay

At a density of 5 × 10^3^ cells per well, MDA-MB-231 and BT549 cells were added to 96-well plates. The putative miRNA binding site of circGNB1 and 3′-UTR of IGF1R was mutated. Constructed plasmids (wild-type or mutant) and miR-141-5p mimics were cotransfected into MDA-MB-231 and BT549 cells for 2 days. Then, luciferase activity was measured by the dual luciferase reporter assay system (Promega) according to the instructions of manufacturer. Independent experiments were conducted in in triplicate.

### RNA Immunoprecipitation (RIP)

The RIP assay for Ago2 was conducted with a Magna RIP RNA-Binding Protein Immunoprecipitation Kit (Millipore, United States), an anti-Ago2 antibody (Millipore, United States) and a normal IgG (Millipore, United States). The level of RNA was quantified after the RNA complexes were purified. The relative abundance of circGNB1, IGF1R and miR-141-5p was determined by qRT-PCR analysis after purification.

### Western Blot Analysis

Total protein of cells was extracted by RIPA lysis buffer, separated by SDS-PAGE and subsequently transferred to PVDF membranes (Millipore). Membranes were blocked with 5% skim milk at room temperature for 1 h and subsequently incubated with the primary antibody anti-IGF1R (1:1000, Abcam, United States) and β-actin antibody (1:1000, Affinity, United States) at 4°C overnight. A secondary antibody (CST) was used and detected by chemiluminescence.

### Mouse Xenograft Model

All animal procedures and care were performed according to the guidelines of the institutes and the approval of the Institute Research Ethics Committee of SYSUCC. MDA-MB-231 and BT549 cells (1 × 10^7^) were subcutaneously injected into the dorsal flanks of female BALB/c nude mice (*n* = 5 for each group) and the mice were treated with an intratumoral injection (50 μL si-NC or si-circGNB1) every 4 days. After 5 weeks, mice were euthanized, and tumors were weighed and recorded. For the lung metastasis experiments, 1 × 10^5^ cells (transfected with si-NC or si-circGNB1) were intravenously injected into the tail vein of mice (*n* = 5 for each group). After 8 weeks, all the mice were euthanized and the lungs were excised. The numbers of lung metastases were counted visually and subsequently confirmed via microscopy of hematoxylin and eosin (HE)-stained sections.

### Statistical Analysis

All the data were analyzed with SPSS 24.0 software (SPSS Inc., Chicago, IL, United States). Quantitative data are presented as the form of mean ± standard deviation (SD). We used two-tailed Student’s *t*-test to compare the difference of two groups. Kaplan-Meier analysis and the log-rank test were implemented to generate the overall survival curves and compare differences between the two cohorts, respectively. *P* < 0.05 was considered statistically significant.

## Results

### circGNB1 Is Upregulated in TNBC and Correlated With Poor Clinical Outcomes

**FIGURE 1:**
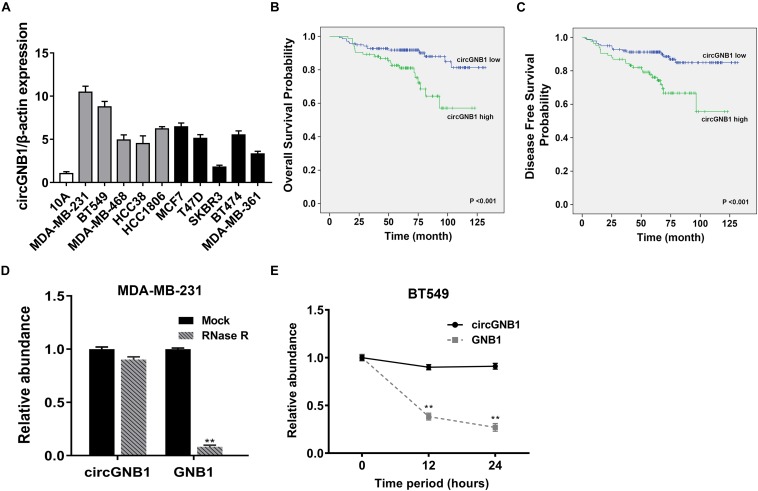
circGNB1 is upregulated in TNBC and correlated with poor clinical outcomes. **(A)** The expression level of circGNB1 in normal mammary cell line MCF-10A and breast cancer cell lines. Gray bar and black bar represent for TNBC and non-TNBC cell lines, respectively. **(B,C)** Kaplan–Meier analysis of the **(B)** overall survival and **(C)** disease-free survival of 222 TNBC patients with circGNB1 high (green) or low (blue) expression levels. **(D)** Relative abundance of circGNB1 and GNB1 mRNA after treatment with RNase R in MDA-MB-231 cells. **(E)** Relative abundance of circGNB1 and GNB1 mRNA after being treated with Actinomycin D in BT-549 cells.

We reanalyzed the circRNA microarray profiling in our previous study (Chen et al., 2018), we founded that hsa_circ_0009362 was frequently upregulated in TNBC tissues compared to the adjacent normal mammary tissues (Supplementary Table S1). By browsing the circBase database and University of California, Santa Cruz (UCSC) Genome Browser, we found that hsa_circ_0009362 is generated from exons 2 and 3 of GNB1 with no intron (chr1:1756835-1770677) which is located on chromosome 1p36.33. Therefore, we named it circGNB1 and designed the divergent primers. By using qRT-PCR analysis, we validated that the expression level of circGNB1 was upregulated in breast cancer cell lines compared to normal mammary cell lines MCF-10A (Figure 1A). To evaluate the prognostic value of circGNB1, a total of 222 patients with TNBC was recruited and divided into two cohorts according to the expression circGNB1 assessed by qRT-PCR analysis. Kaplan-Meier survival analysis showed that high expression level of circGNB1 was associated with a poor overall survival (OS) and disease-free survival (DFS) outcomes (Figures 1B,C). To investigate the correlation between the circGNB1 expression level and clinicopathological characteristics in TNBC, we did further statistical analysis. The expression of circGNB1 was positively correlated with tumor size and clinical stage, and high expression of circGNB1 was an independent risk factor for TNBC patients (Tables 1, 2). RNase R digestion experiment and Actinomycin D assay was conducted to verify the circular characteristics of circGNB1 in MDA-MB-231 and BT549, respectively (Figures 1D,E).

**TABLE 1:** Correlation of circGNB1 expression with clinicopathologic characteristics of triple-negative breast cancer patients.

**Variables**	**Cases**	**circGNB1**		***P*-value**
		**Low**	**High**	
**Age (*y*)**				
>50	132	80(60.6%)	52(39.4%)	
≤50	90	58(64.4%)	32(35.6%)	0.563
**Menopause**				
Yes	132	81(61.4%)	51(38.6%)	
No	90	57(63.3%)	33(36.7%)	0.766
**Tumor size**				
≤2.0 cm	59	50(84.7%)	9(15.3%)	
>2.0 cm	163	88(54.0%)	75(46.0%)	0.001*
**Lymph node status**				
Negative	107	73(68.2%)	34(31.8%)	
Positive	115	65(56.5%)	50(43.5%)	0.072
**TNM stage**				
I-II	172	115(66.9%)	57(33.1%)	
III-IV	50	23(46.0%)	27(45.0%)	0.007*

*P < 0.05, statistically significant.

**TABLE 2:** Univariate and multivariate Cox regression analysis of circGNB1 and survival in patients with triple-negative breast cancer.

**Parameter**	**Univariate analysis**			**Multivariate analysis**		
		
	**HR**	**95% CI**	***P*-value**	**HR**	**95% CI**	***P*-value**
Age (>50 vs. ≤50 years)	0.632	0.304–1.312	0.219	NA		
Menopause (Yes vs. No)	0.670	0.329–1.362	0.258	NA		
Histological grade (G3 vs. G1-2)	1.434	0.740–2.782	0.286	NA		
Tumor size (>2.0 cm vs. ≤2.0 cm)	3.141	1.110–8.889	0.013*	2.072	0.708–6.064	0.184
Lymph node status (Positive vs. Negative)	2.807	1.350–5.836	0.006*	1.514	0.645–3.553	0.340
TNM stage (III–IV vs. I–II)	3.365	1.740–6.510	0.001*	2.703	1.360–5.373	0.005*
circGNB1 expression (High vs. Low)	2.759	1.409–5.404	0.003*	2.148	1.070–4.310	0.031*

NA: not analyze; *P < 0.05, statistically significant.

### Downregulation of circGNB1 Suppresses the Proliferation and Metastasis of TNBC Cells *in vitro*

**FIGURE 2:**
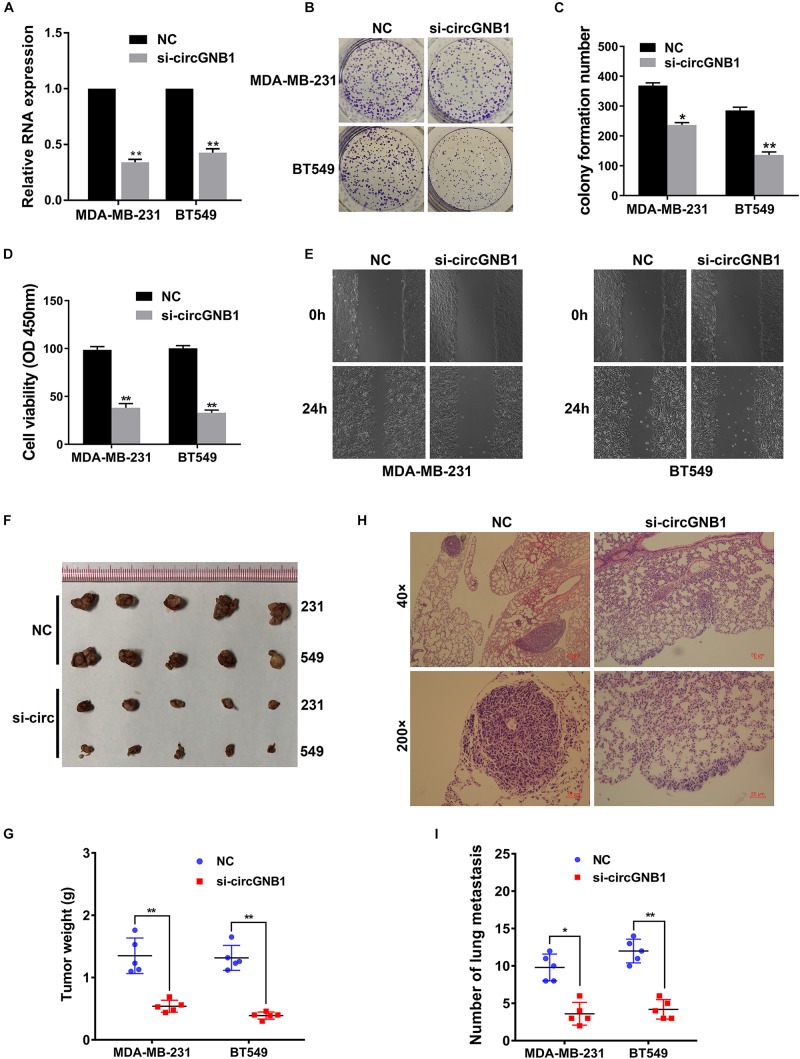
Downregulation of circGNB1 suppresses the proliferation and metastasis of TNBC cells *in vitro*. **(A)** Efficacy of siRNA targeting circGNB1 was assessed by qRT-PCR analysis. **(B,C)** circGNB1 inhibits the colony forming ability of MDA-MB-231 and BT549 cells. **(D)** Cell proliferation was detected by CCK-8 assays. **(E)** Wound-healing assays assessed the impact of circGNB1 on cell migration ability. **(F)** Mouse xenograft models were established. **(G)** Tumor weight were measured and recorded. **(H)** Hematoxylin-eosin staining was conducted and sections of lung metastases are showed. **(I)** The number of lung metastases was counted and recorded. *P < 0.05; **P < 0.01.

To investigate whether circGNB1 was involved in the progression of TNBC, we next performed loss-of-function assays. We designed siRNA targeting the back-splicing region of circGNB1 and validated its efficacy by qRT-PCR analysis (Figure 2A). qRT-PCR analysis showed that the siRNA could only target the back-splice junction of circRNA and have no effect on linear GNB1 mRNA expression (Supplementary Figure S1A). Next, we conducted CCK-8 assays and colony formation assays to assess the influence of circGNB1 on cell proliferation and the ability of colony-forming. We found that knock down of circGNB1 could significantly inhibit the growth and colony-forming ability in MDA-MB-231 and BT-549 cell lines (Figures 2B,C). Wound healing assay revealed that silencing of circGNB1 markedly reduced the migration ability of these two TNBC cell lines (Figure 2E and Supplementary Figure S1B). To further assess the biological roles of circGNB1 in vivo, mouse xenograft models were established. Consistent with the findings in cell experiments, downregulation of circGNB1 could reduce the tumor volume (Figures 2F,G) and decrease the total number of lung metastases (Figures 2H,I). These results demonstrated the pro-cancerous function of circGNB1 in TNBC.

### circGNB1 Functions as a Sponge of miR-141-5p

**FIGURE 3:**
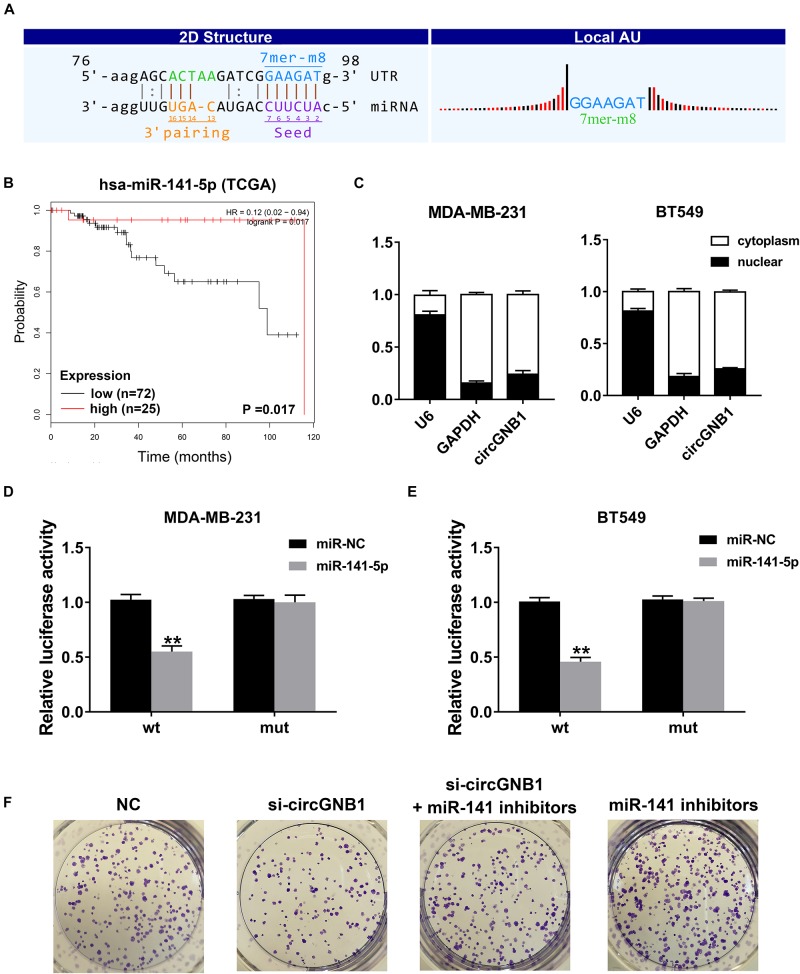
circGNB1 functions as a sponge of miR-141-5p. **(A)** Predicted binding site of miR-141-5p within circGNB1 according to MRE. **(B)** Kaplan-Meier analysis of the association between miR-141-5p and overall survival in patients with triple negative breast cancer from TCGA public online database (http://kmplot.com/analysis). **(C)** U6 (nuclear control transcript), GAPDH (cytoplasmic control transcript) and circGNB1 in nuclear and cytoplasmic fractions analyzed by qRT-PCR. **(D–E)** Luciferase reporter assays of MDA-MB-231 and BT549 cells co-transfected with miR-141-5p mimics and circGNB1 wild type or mutant luciferase reporter plasmid. **(F)** The colony formation ability enhanced by miR-141-5p inhibitors were reversed after co-transfected with si-circGNB1 using colony formation assay. *P < 0.05; **P < 0.01.

Given that circRNA has been proven to be a miRNA sponge in multiple cancers, we next predicted the potential binding miRNA of circGNB1 to elucidate the underlying molecular mechanism. According to miRNA response elements (MREs) analysis, miR-141-5p was predicted to have the potential to interact with circGNB1 (Figure 3A). By analyzing the miRNA microarray data derived from TCGA, we found that high expression of miR-141-5p was associated with better OS in TNBC patients^1^ (Figure 3B). According to the reported research, downregulation of miR-141-5p was associated with progression and trastuzumab resistance in breast cancer (Finlay-Schultz et al., 2015; Li et al., 2017; Han et al., 2019). Detected by qPCR analysis, miR-141-5p was downregulated in TNBC cell lines compared to that in mammary epithelial cell lines (Supplementary Figure S2A). Additionally, detected by qRT-PCR analysis, circGNB1 was predominantly existed in the cytoplasm where miRNAs were mostly located in Figure 3C. Therefore, we conducted dual luciferase reporter assays to determine the interaction between miR-141-5p and circGNB1. We cotransfected a full-length of circGNB1-wild-type (WT) or a circGNB1-mutant (mutation of putative miRNA binding site) luciferase reporter plasmid with miR-141-5p mimics or control mimics into MDA-MB-231 and BT549 cells. The results revealed that miR-141-5p mimics could reduce the relative luciferase activity of the WT reporter but not the mutant reporter (Figures 3D,E). Moreover, clone-formation assays indicated that the blockage of miR-141-5p significantly enhanced the colony-forming ability of BT549 cells, and this effect could be reversed by circGNB1 silencing (Figure 3F and Supplementary Figure S2B).

### circGNB1 Promotes TNBC Cell Growth and Proliferation via circGNB1-miR-141-5p-IGF1R Axis

**FIGURE 4:**
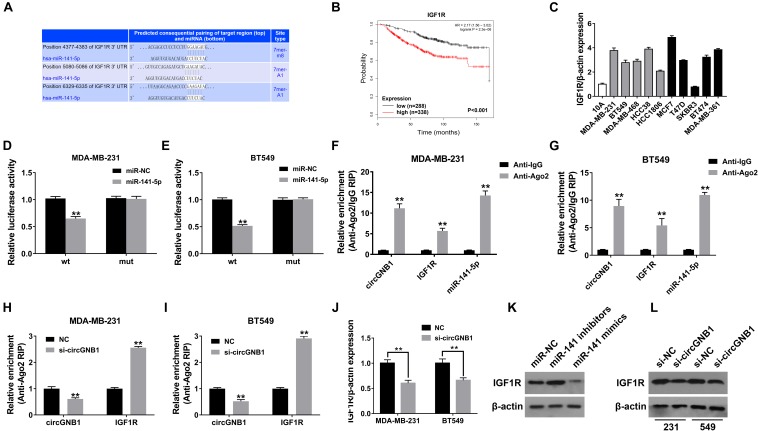
circGNB1 promotes TNBC cell growth and proliferation via circGNB1-miR-141-5p-IGF1R axis. **(A)** TargetScan algorithm was used to predict binding sites of miR-141-5p within the 3′-UTR of IGF1R mRNA (http://www.targetscan.org). **(B)** Kaplan-Meier analysis of the association between miR-141-5p and overall survival in patients with breast cancer from public online database (http://kmplot.com/analysis). Two probes were used in this analysis (225330_at and 243358_at). **(C)** The relative expression level of IGF1R in breast cancer cell lines. Gray bar and black bar represent for TNBC and non-TNBC cell lines, respectively. **(D,E)** Luciferase reporter assay of MDA-MB-231 and BT549 cells co-transfected with miR-141-5p mimics and the 3′-UTR of IGF1R wild type or mutant luciferase reporter. **(F,G)** Enrichment of circGNB1, IGF1R and miR-141-5p on Ago2 assessed by RIP assay. **(H,I)** The enrichment of circGNB1 was decreased, while IGF1R expression was increased after knockdown of circGNB1 assessed by RIP assay. **(J)** Expression of IGF1R was decreased after transfection with si-circGNB1 detected by qPCR. **(K)** Expression of IGF1R was assessed by western bolt analysis after transfected with miR-141-5p mimics or inhibitors. **(L)** The impact of knockdown of circGNB1 on IGF1R protein expression in MDA-MB-231 and BT549 cells. *P < 0.05; **P < 0.01.

To identify the downstream targets of miR-141-5p, TargetScan algorithm was used to predicted potential oncogenes. Among these candidate genes, IGF1R was predicted which has been confirmed as a robust oncogene in breast cancer, including TNBC (Klinakis et al., 2009; Castano et al., 2013; Obr et al., 2018; Figure 4A). By analyzing the public online database, high expression level of IGF1R (probe: 225330_at and 243358_at) was associated with poor OS in patients with breast cancer, which was consisted with the previously reported study (Figure 4B). Detected by qPCR analysis, IGF1R was upregulated in breast cancer cell lines compared mammary epithelial cell lines (Figure 4C). Subsequently, dual luciferase reporter assays were carried out to confirm the binding between miR-141-5p and the 3′-UTR of IGF1R transcription. The results showed that the luciferase density was decreased after cotransfection with miR-141-5p mimics and the WT-3′-UTR IGF1R plasmid, compared to the mutant 3′-UTR IGF1R reporter in both MDA-MB-231 and BT549 cells (Figures 4D,E). In addition, RIP assays were performed to further demonstrate the direct interaction between miR-141-5p and IGF1R mRNA. circGNB1, IGF1R, and miR-141-5p were predominantly enriched on Ago2 which is the component of RNA-induced silencing complex (RISC) (Figures 4F,G). The enrichment of circGNB1 was decreased, while IGF1R expression was increased after knockdown of circGNB1 assessed by RIP assay (Figures 4H,I). Inhibition or overexpression of miR-141-5p could increase or decrease the expression of IGF1R determined by western blot analysis (Figure 4J). Furthermore, the result of qRT-PCR and western blot analysis revealed that si-circGNB1 could not only reduce the mRNA level of IGF1R but also change its protein expression level (Figures 4K,L).

## Discussion

As an intriguing class of RNA, circRNAs have attracted tremendous attention of the researchers and become one of the hottest topics in the field of biomedicine. Compared to linear RNAs, circRNAs are characterized for their covalently closed structure with no head or tail (Li et al., 2018). Advances made in novel bioinformatics algorithms and high-throughput sequencing technology make it easier for scientists to detect and identify circRNAs (Salzman et al., 2013). Several circRNA databases were established to identify and characterized thousands of circRNAs, including CircBase (Glazar et al., 2014), CIRCpedia (Zhang et al., 2016) and MiOncoCirc database (Vo et al., 2019). In recent years, an increasing number of circRNAs have been identified and well-studied in the cancer research. As the most well-known circRNA, CDR1as (also termed as ciRS-7) was firstly uncovered as a mediator of biological processes containing over 70 conventional binding sites for miR-7 (Hansen et al., 2013a). CDR1as/ciRS-7 was found to be an oncogenic molecule which promotes proliferation, metastasis and may regulate the tumor microenvironment of multiple cancers by sponging miR-7 (Hansen et al., 2013b; Weng et al., 2017; Pan et al., 2018; Zou et al., 2019a). Similarly, circRNA can also act as a tumor suppressor in the development and progression of cancer. For example, circFBXW7 inhibits tumor growth and metastasis in glioma and breast cancer by encoding a 21kDa novel protein FBXW7-185aa and blocking miR-197-3p (Yang et al., 2018; Ye et al., 2019). circMTO1 suppresses malignancy progression by serving as the sponge of oncogenic miR-9 to upregulate p21 expression in human hepatocellular carcinoma (Han et al., 2017). However, the functions and roles of the majority of circRNAs are still remained unknown.

In the current study, we validated that circGNB1 was overexpressed in TNBC cell lines and high expression of circGNB1 was associated with worse clinical features and survival outcomes. The expression of circGNB1 was positively correlated with tumor size and clinical stage, and high expression of circGNB1 was an independent risk factor for TNBC patients. Both *in vitro* and *in vivo* functional assays revealed that knock down of circGNB1 significantly suppressed cell proliferation, migration and tumor growth. Further mechanically experiments showed that circGNB1 sponges miR-141-5p and inhibits TNBC progression by upregulating oncogene IGF1R expression. According to the bioinformatic analysis and public database, miR-141-5p was predicted as the downstream of circGNB1 and low expression of miR-141-5p was associated with an improved overall survival clinical outcome. Downregulation of miR-141 contributes to the cancer cell growth, migration, trastuzumab resistance and stem cell expansion in breast cancer (Finlay-Schultz et al., 2015; Li et al., 2017; Han et al., 2019). IGF1R is a receptor binding insulin-like growth factor with a high affinity with tyrosine kinase activity, which is highly overexpressed in most malignant tissues and enhances cell survival (Pollak, 2008). IGF1R signaling can stimulate cell proliferation and protect cell from stress in triple negative breast cancer and is regarded as a therapeutic target for treatment (Klinakis et al., 2009; Castano et al., 2013; Obr et al., 2018). We found that knockdown of circGNB1 can significantly decrease the expression of IGF1R indicating that circGNB1 could become a potential target for the inhibition of TNBC. All these results demonstrated the pivotal role of circGNB1 and its prognostic value for TNBC patients.

Lacking of effective therapeutic target, TNBC is the breast cancer subtype with the highest metastatic rate and worst prognosis. Metastasis is the main cause of mortality in patients with breast cancer, which accounts for over 90% of the death cases (Arnedos et al., 2015). Metastatic TNBC is unresectable and the treatment is limited to chemotherapy and burgeoning immunotherapy with a general therapeutic efficacy and frequent adverse effect (Santa-Maria and Gradishar, 2015; Schmid et al., 2018; Xiao et al., 2018b). Decryption of circGNB1 may provide new strategies for TNBC therapy or diagnosis. Thus, delivery of siRNA may also become an ideal strategy for TNBC treatment in the near future.

In conclusion, our study identified circGNB1 as an oncogenic molecule and demonstrated the pivotal role of circGNB1-miR-141-5p-IGF1R axis in TNBC growth and metastasis. Therefore, circGNB1 may have the potential to be a therapeutic target and novel prognostic biomarker for TNBC.

## Data Availability Statement

The raw data supporting the conclusions of this article will be made available by the authors, without undue reservation, to any qualified researcher.

## Ethics Statement

This study was approved by the Ethics Committee of Sun Yat-sen University Cancer Center Health Authority and conducted in accordance with the Declaration of Helsinki. Written informed consent was collected from all patients before participation in this study.

## Author Contributions

YK and WW designed the experiments and reviewed and revised the manuscript. PL, XL, and JZ performed the experiments. PL, AY, and FY analyzed and interpreted the data. YZ and PL was the major contributors in writing the manuscript. All authors read and approved the final version of the manuscript.

## Conflict of Interest

The authors declare that the research was conducted in the absence of any commercial or financial relationships that could be construed as a potential conflict of interest.

## Footnotes

1 http://kmplot.com/analysis/

## Supplementary Material

FIGURE S1 Downregulation of circGNB1 suppresses the proliferation and metastasis of TNBC cells *in vitro*. **(A)** Expression of linear GNB1 mRNA was evaluated by qRT-PCR analysis after transfection with si-circGNB1. **(B)** Statistical graph of the wound-healing assays.

FIGURE S2 circGNB1 functions as a sponge of miR-141-5p. **(A)** The relative expression level of miR-141-5p in breast cancer cell lines. Gray bar and black bar represent for TNBC and non-TNBC cell lines, respectively. **(B)** Statistical graph of the colony formation assay.

TABLE S1 The full array data of the circRNA.

TABLE S2 The sequences of qRT-PCR primers used in this study.

## Abbreviations

3′-UTR: 3′-untranslated region
ceRNA: competing endogenous RNAs
circRNA: circular RNA
IGF1R: insulin like growth factor 1 receptor
miRNAs: microRNAs
RIP: RNA immunoprecipitation
SD: standard deviation
siRNA: small interfering RNA
TNBC: triple-negative breast cancer.
