# Supplementary figures and images for: circGNB1 Facilitates Triple-Negative Breast Cancer Progression by Regulating miR-141-5p-IGF1R Axis
Source: Front Genet. 2020 Mar 5;11:193. doi: 10.3389/fgene.2020.00193 (PMC7066119; doi:10.3389/fgene.2020.00193)

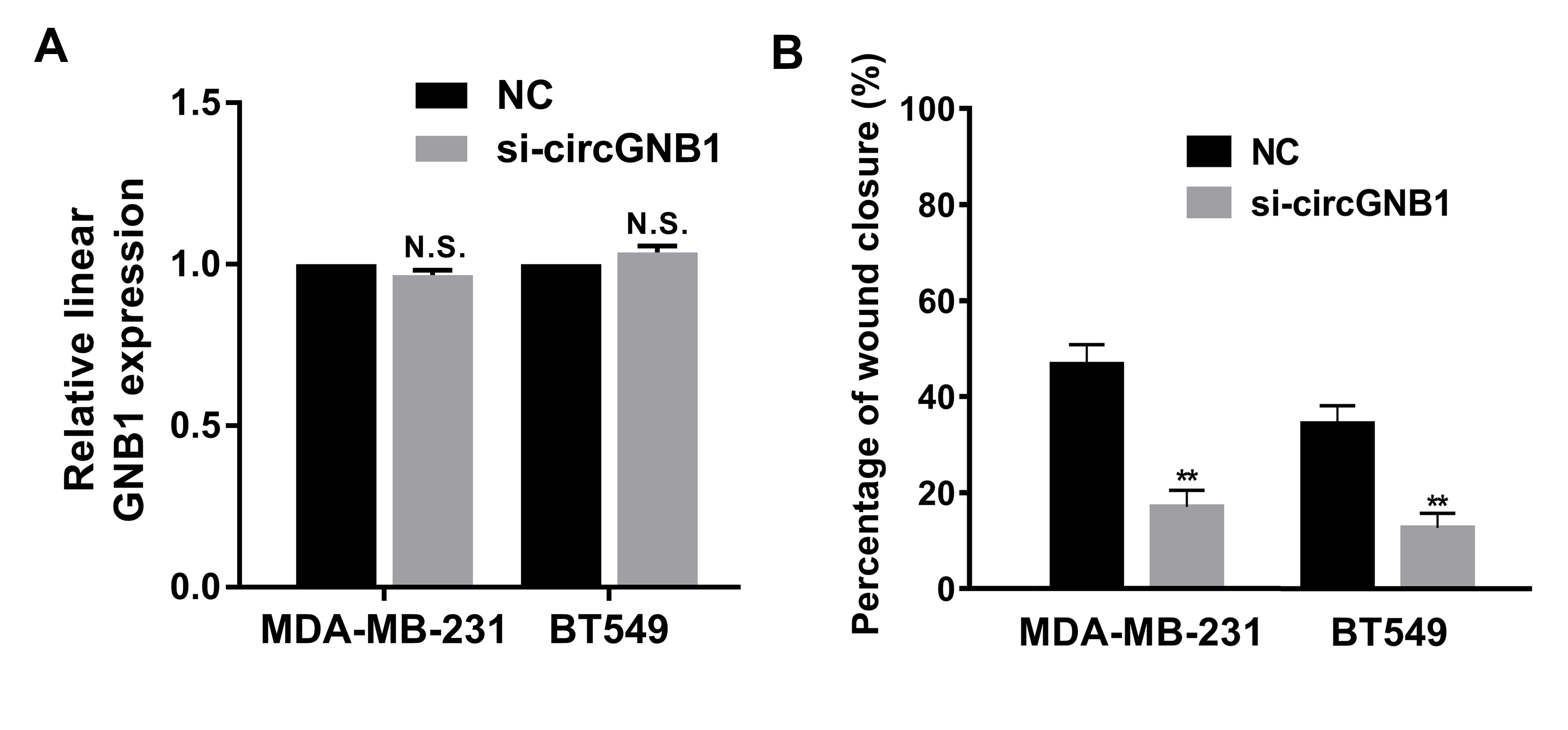

Supplement: FIGURE S1 — Downregulation of circGNB1 suppresses the proliferation and metastasis of TNBC cells in vitro. (A) Expression of linear GNB1 mRNA was evaluated by qRT-PCR analysis after transfection with si-circGNB1. (B) Statistical graph of the wound-healing assays. [file Image_1.TIF]

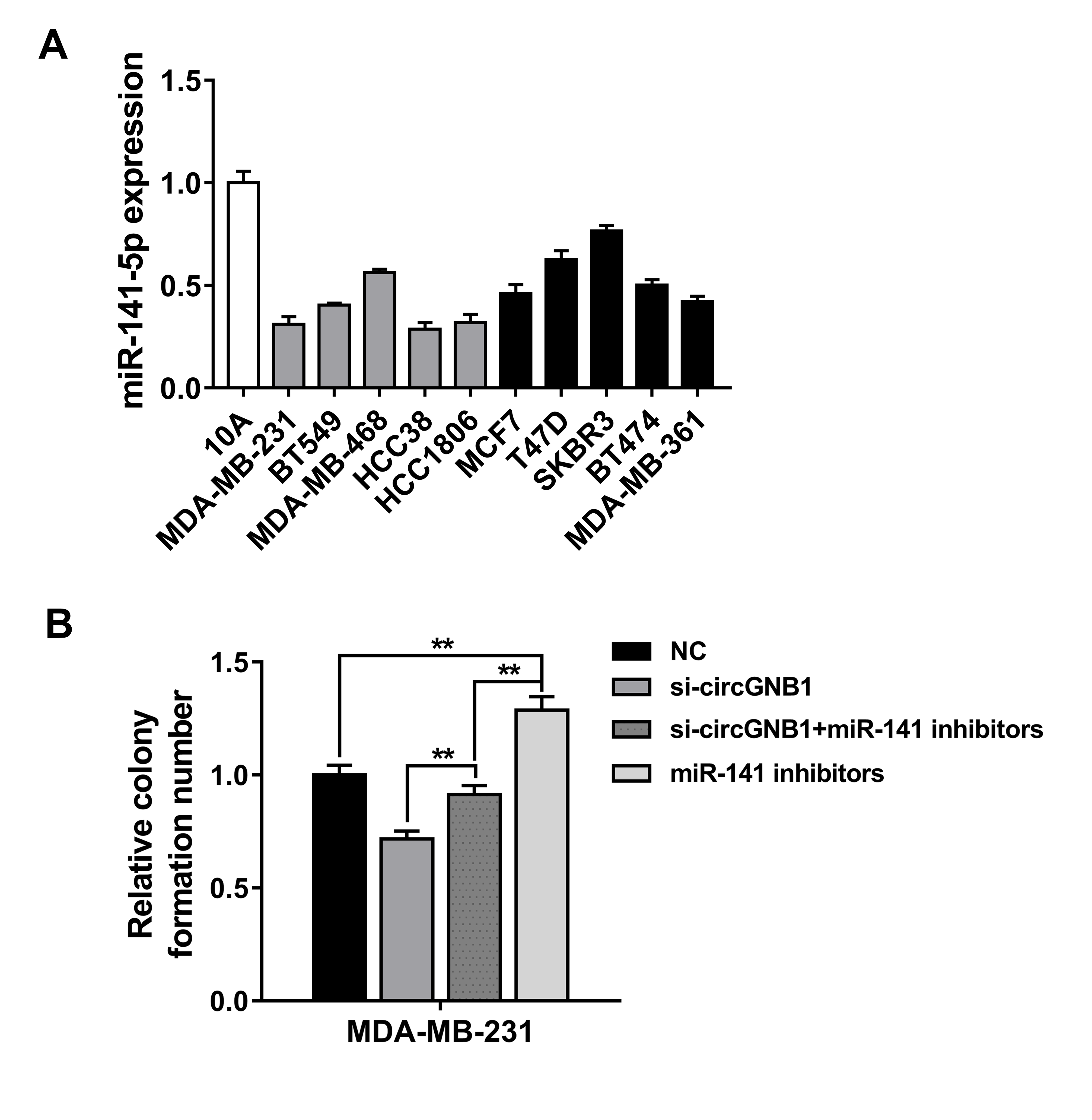

Supplement: FIGURE S2 — circGNB1 functions as a sponge of miR-141-5p. (A) The relative expression level of miR-141-5p in breast cancer cell lines. Gray bar and black bar represent for TNBC and non-TNBC cell lines, respectively. (B) Statistical graph of the colony formation assay. [file Image_2.TIF]
