# Supplementary material for: circGNB1 Facilitates Triple-Negative Breast Cancer Progression by Regulating miR-141-5p-IGF1R Axis
Source: Front Genet. 2020 Mar 5;11:193. doi: 10.3389/fgene.2020.00193 (PMC7066119; doi:10.3389/fgene.2020.00193)
Supplement: TABLE S1 — The full array data of the circRNA. [file Table_1.docx]

Tables S1. The sequences of qRT-PCR primers used in this study.

| **qRT-PCR Primers (**5’→3’**)** | |
| --- | --- |
| circGNB1-F | CTTAAGAACCAGATTCGAACAAAT |
| circGNB1-R | TGGTCAAGCTCACTCATCTT |
| GNB1-F | GAAGATGAGTGAGCTTGACCAG |
| GNB1-R | CATGCTTTCCTGGCGTCT |
| IGF1R-F | TTCAGCGCTGCTGATGTG |
| IGF1R-R | GGCTCATGGTGATCTTCTCC |
| miR-141-5p-F | GTCGTATCCAGTGCAGGG |
| miR-141-5p-R | CGACGTAACACTGTCTGG |
| β-actin-F | GAAATCGTGCGTGACATTAA |
| β-actin-R | AAGGAAGGCTGGAAGAGTG |
| GAPDH-F | GCACCGTCAAGGCTGAGAAC |
| GAPDH-R | TGGTGAAGACGCCAGTGGA |
| U6-F | CTCGCTTCGGCAGCACA |
| U6-R | AACGCTTCACGAATTTGCGT |
